# Large Cervical Leiomyoma of the Uterus: A Rare Cause of Chronic Pelvic Pain Associated With Obstructive Uropathy and Renal Dysfunction: A Case Report

**DOI:** 10.7759/cureus.33387

**Published:** 2023-01-05

**Authors:** Efthymia Thanasa, Anna Thanasa, Evangelos Kamaretsos, Ioannis Paraoulakis, Apostolos Ziogas, Gerasimos Kontogeorgis, Vasiliki Grapsidi, Ektoras-Evangelos Gerokostas, Vasileios Kontochristos, Ioannis Thanasas

**Affiliations:** 1 Department of Histology, Department of Health Sciences, Medical School, Aristotle University of Thessaloniki, Thessaloniki, GRC; 2 Department of Anatomy, Department of Health Sciences, Medical School, Aristotle University of Thessaloniki, Thessaloniki, GRC; 3 Department of Obstetrics and Gynecology, General Hospital of Trikala, Trikala, GRC; 4 Department of Obstetrics and Gynecology, University of Thessaly, Larissa, GRC

**Keywords:** cervical leiomyoma, hydronephrosis, renal dysfunction, imaging studies, surgical treatment, case report

## Abstract

Large cervical leiomyomas (≥10cm) are extremely rare. Our case report concerns the surgical treatment of a patient with a large cervical leiomyoma associated with chronic pelvic pain, bilateral hydroureteronephrosis and significant impairment of renal function. A 47-year-old patient of reproductive age with a normal menstrual cycle and a medical history of chronic pelvic pain presented to the gynecology clinic for examination. Clinically, the presence of a large pelvic mass was found, the upper margins of which were palpable at the level of the umbilicus. A preoperative assessment revealed bilateral hydroureteronephrosis due to obstructive uropathy and renal dysfunction. Hydroureteronephrosis, as a consequence of the large pelvic mass, probably originating from the cervix of the uterus, was evaluated as the main cause of renal dysfunction. Tumor markers were negative. The imaging studies confirmed the clinical diagnosis of uterine leiomyoma, and the surgical treatment of the patient with laparotomy was decided. Intraoperatively, the presence of a large uterine cervical fibroid was detected, and a total abdominal hysterectomy and bilateral adnexectomy were performed. Operating was difficult, with significant surgical difficulties. The postoperative course was uneventful, without immediate complications. The patient's symptom relief began gradually, immediately after surgery. Three months after surgery, the patient reported complete relief of her pelvic pain. A re-examination of the urinary tract revealed complete recovery of renal morphology and function. In the paper, after the presentation of the case, a brief review of cervical leiomyomas is attempted based on the literature, mainly regarding the diagnostic and therapeutic approach.

## Introduction

Leiomyomas, also known as fibroids, are the most common uterine neoplasms. They are benign estrogen-dependent tumors consisting mainly of myometrial smooth muscle cells. Fibroids usually occur during reproductive age and are more common in women of African descent than white women [1]. The etiopathogenetic mechanism has not been fully clarified. Age, origin, family history, reproductive health issues, sexual characteristics, certain dietary habits, and estrogen seem to play an important role in the pathogenesis of the disease [2]. The insufficient understanding of the molecular mechanisms that initiate or promote the pathogenesis of uterine leiomyomas and the lack of preclinical models that underlie the molecular environment of the disease makes it impossible to date to have an effective treatment option that will be long-term, cost-effective, and fertility-sparing [3]. Uterine fibroids, according to their anatomical location, are divided into intramural, subserosal, and submucosal. A significant increase in the size of subserosal and submucosal fibroids implies the appearance of pedunculated uterine tumors [4]. In uncommon cases, as in our patient, the localization of the fibroid in the female reproductive tract may involve the cervix.

The current case report emphasizes the significant degree of bilateral hydroureteronephrosis with deterioration of renal function and the significant surgical difficulties that may arise when treating rare cases of large leiomyomas originating from the cervix. At the same time, it is pointed out that, despite their rarity, large uterine cervical leiomyomas must be included among other pathological conditions in the differential diagnosis of chronic pelvic pain associated with hydroureteronephrosis, and deterioration of renal function in women of reproductive age, to avoid permanent renal parenchymal disease and to ensure the good health and quality of life of these patients.

## Case presentation

**Figure 1:**
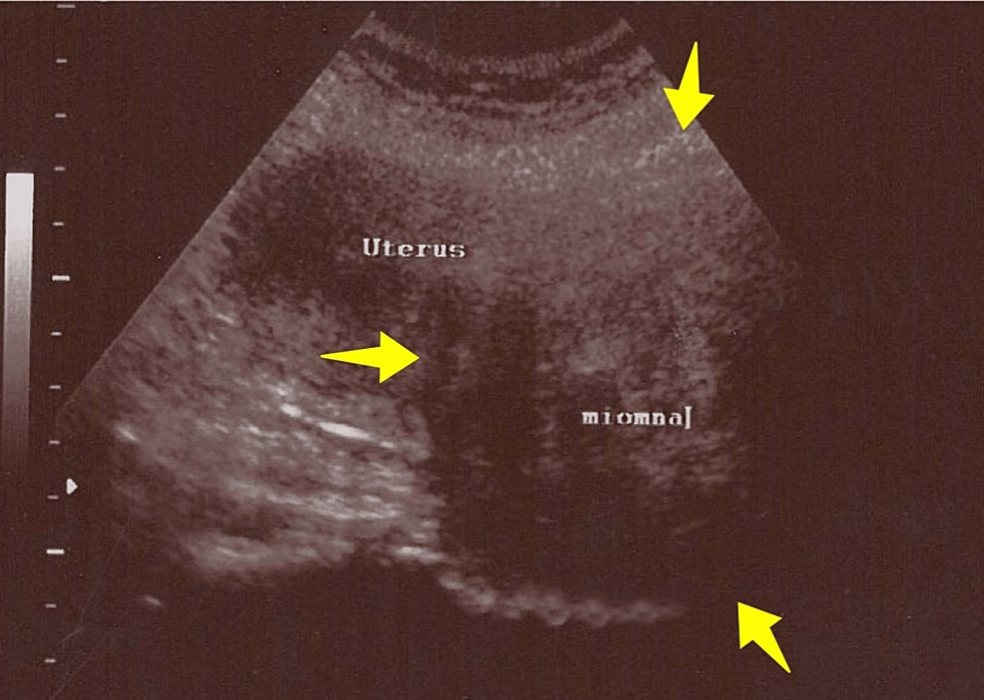
Transabdominal ultrasound imaging of uterine cervical leiomyoma (our case) In the anatomical position of the cervix, the demarcation of a space-occupying lesion (yellow arrows), several times the size of the uterine corpus, is evident.

A 47-year-old patient with a normal menstrual cycle came for a gynecological examination. The patient had two vaginal deliveries in her obstetric history and a medical history of hyperlipidemia and arterial hypertension, well-regulated with medication. From the history, neither chronic kidney disease was reported, nor recurrent urinary tract infections in recent years. From the personal medical history, the presence of pelvic pain during the last two years, accompanied by occasional mild episodes of frequent urination and dysuria, was mainly reported. The clinical examination revealed the presence of a large pelvic mass, the upper margins of which were palpable at the level of the umbilicus. Transabdominal ultrasound disclosed a solid, discrete, echogenic mass occupying the entire pelvic cavity, likely originating from the cervix (Figure 1).

**Figure 2:**
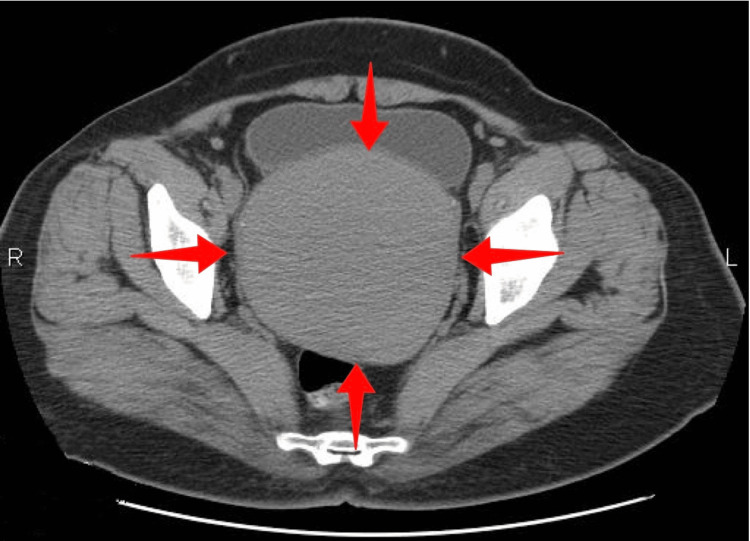
Computed tomography imaging of uterine cervical leiomyoma (our case) The pelvic mass depicted within the red arrows corresponds to a large cervical leiomyoma.

Computed tomography confirmed the ultrasound findings but could not rule out the presence of a pedunculated subserosal uterine fibroid or a solid adnexal mass (Figure 2).

**Figure 3:**
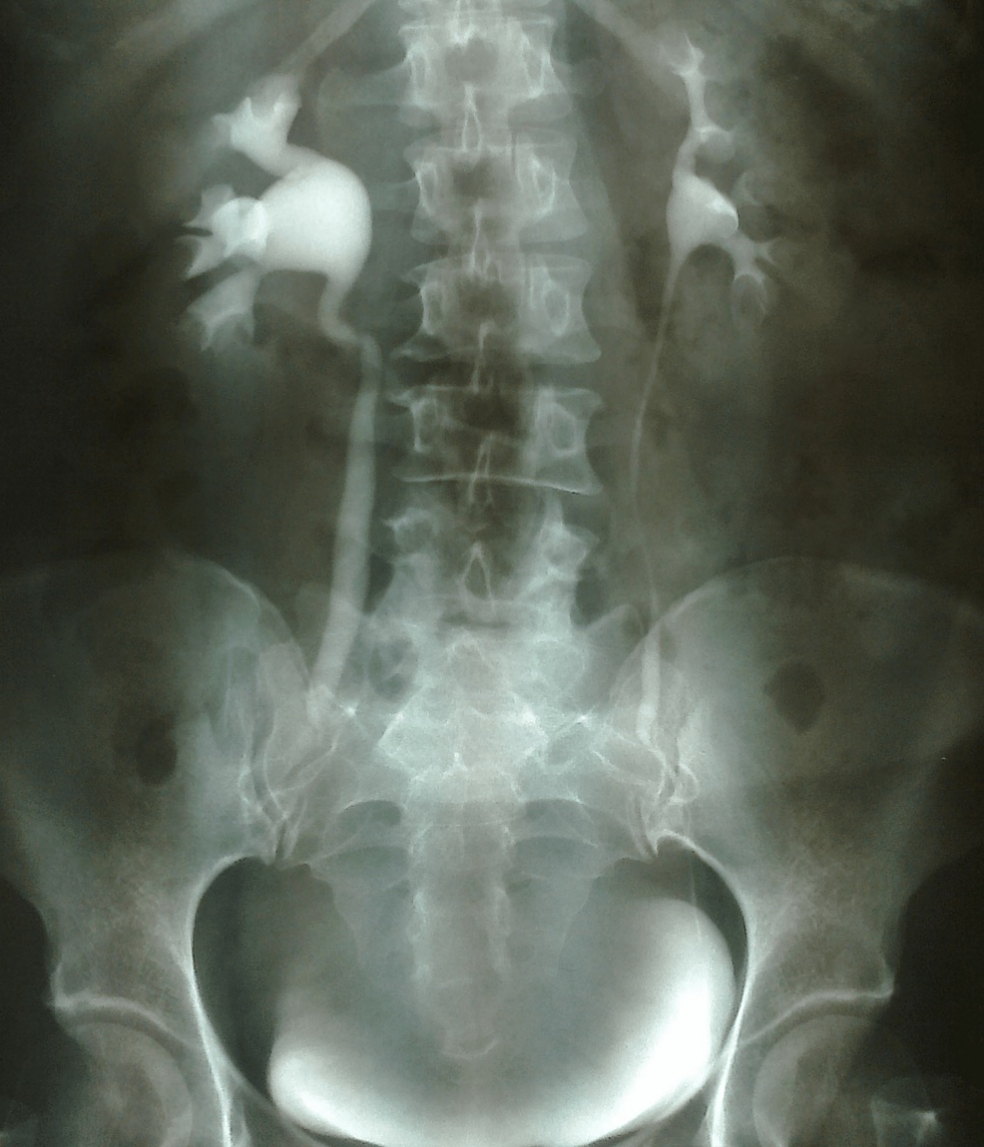
Preoperative imaging of the urinary tract by intravenous pyelography in a patient with a large cervical leiomyoma of the uterus (our case) Bilateral hydroureteronephrosis, displacement of the dilated ureters, and incomplete bladder filling are evident.

Intravenous pyelography revealed bilateral dilatation of the pelvicalyceal system, displacement of the ureters, and incomplete filling of the bladder (Figure 3).

The laboratory analysis upon patient admission to our clinic found: Ht 39.6%, Hb 13.1gr/dl, PLT 260x103/ml, U 83mg/dl, Cr 1.9mg/dl, Na 142mEq/lt, K 4.2mEq/lt. Inflammatory markers were negative. The urine culture was without evidence of urinary tract infection. Cytology (Test Papanikolaou) of the displaced and partially deformed cervix by the pelvic mass, which took place preoperatively, was negative for malignancy. Tumor markers (CEA, Ca125, Ca15-3, Ca19-9) were within normal range. The examination by the physicians of the nephrology clinic of our hospital did not find any comorbidity that could be associated with impaired renal function.

The clinical diagnosis of uterine leiomyoma was confirmed by imaging, and it was decided to treat the patient surgically with laparotomy after the pre-operative insertion of pigtail catheters. After opening the abdominal wall and peritoneum, the presence of a large cervical fibroid was detected and a total abdominal hysterectomy with bilateral adnexectomy was performed. After a technically difficult surgery accompanied by significant blood loss, intraoperative transfusion with packed red blood cells was deemed necessary. Transfusion with second-packed red blood cells was also assessed as necessary on the first postoperative day to stabilize the patient hemodynamically. Histological examination of the surgical specimen confirmed the intraoperative diagnosis: the body of the uterus was strongly deformed by the presence of a large leiomyoma in the cervix with a maximum diameter of 14cm. Increased cellularity, coagulative necrosis, atypia, or increased number of nucleokinesis was not observed.

After a smooth postoperative course and immediate undisputed improvement of renal function (serum creatinine 1.5mg/dl), the patient was discharged from our clinic on the fifth postoperative day. Three months later, the patient was completely free of chronic pelvic pain and its accompanying symptoms.

**Figure 4:**
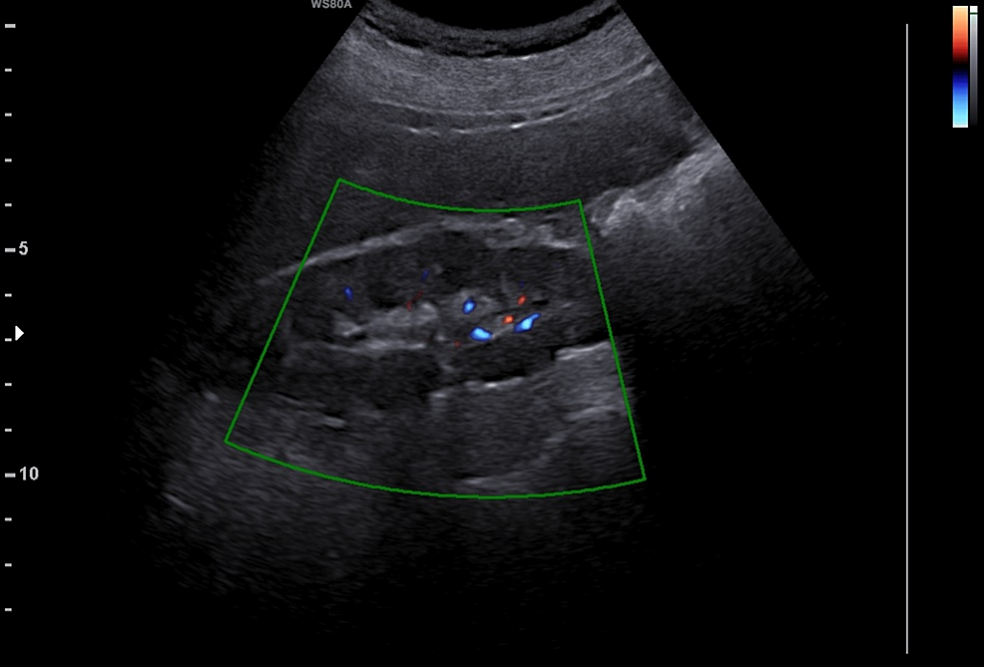
Postoperative ultrasound imaging of the right kidney (our case) The absence of the preoperative dilatation of the pelvicalyceal system and the presence of adequate perfusion of the renal parenchyma is evident.

**Figure 5:**
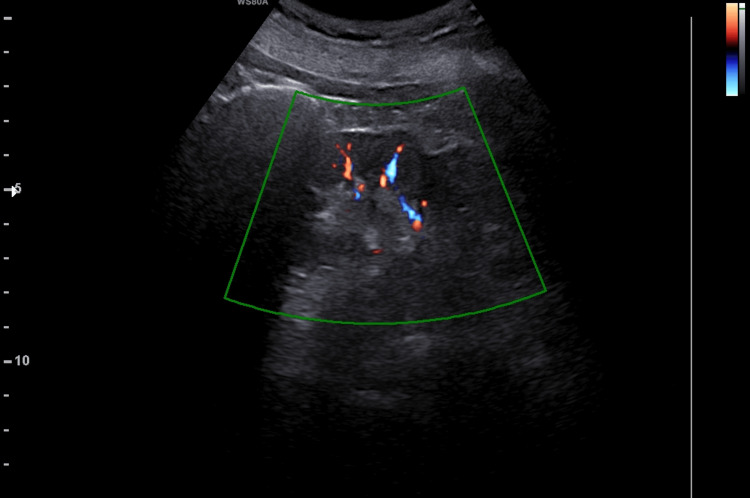
Postoperative ultrasound imaging of the left kidney (our case) The absence of the preoperative dilatation of the pelvicalyceal system and the presence of adequate perfusion of the renal parenchyma is evident.

The ultrasound revealed a complete remediation of the renal morphology and function. The kidneys were visualized with normal echogenicity, normal perfusion, and without dilation of the pelvicalyceal system (Figures 4, 5). Serum creatinine was 0.8mg/dl. To this day, the patient remains under regular follow-up at the Nephrology and Gynecology outpatient clinic of our hospital.

## Discussion

Cervical leiomyomas are located on the cervix. Cervical leiomyomas are divided into two types depending on their localization: the extra cervical type, which is characterized by the growth of the tumor outside the cervix, and the intracervical type (our case), whose fibroids grow inside the cervical lumen [5,6]. Cervical fibroids are rare. It is estimated that they represent approximately 0.6% of the total uterine fibroids, while large cervical fibroids (≥10 cm) are even rare [7]. Extremely rare isolated cases of pedunculated cervical fibroid prolapse during pregnancy that were successfully treated surgically have been reported in the literature [8]. Also, it should not be confused with cervical adenomyoma, which today is a separate nosological entity. It is a rare benign lesion located in the cervix that is characterized by a localized form of adenomyosis which is surrounded by benign muscle tissue, forming a tumor similar to that of cervical leiomyoma [9].

The clinical diagnosis of uterine cervical fibroids is challenging. These patients, when the fibroids are small, are usually asymptomatic. Abnormal uterine bleeding, chronic pelvic pain, dysmenorrhea, dyspareunia, and infertility are the main clinical manifestations that characterize symptomatic patients with cervical uterine leiomyomas [10,11]. In many cases, however, the symptoms are unclear and non-specific. These patients usually complain of frequent urination, abdominal distention, and pain in the lower abdomen in the form of chronic pelvic pain, while they rarely report menstrual disorders [10]. So, it was not surprising that our patient reported no symptoms related to menstruation, except for chronic pelvic pain and occasional mild episodes of frequent urination and dysuria. The appearance of severe pressure symptoms from the rectum and bladder with acute urine retention due to urethral obstruction or urinary incontinence accompanied by hydroureteronephrosis characterize the presence of large cervical fibroids [12,13]. The unusual thing about our patient was that despite the large size of the leiomyoma (14 cm), she did not present severe clinical pressure phenomena from the rectum or the bladder. Our patient did not report constipation, urinary incontinence, or urinary retention. Significant bilateral hydroureteronephrosis and deteriorated renal function were found incidentally during the preoperative screening of the pelvic mass.

In contrast to clinical criteria, imaging can be instrumental in delineating the location and quality of various space-occupying lesions originating from the cervix. On its classical ultrasound imaging, cervical leiomyoma corresponds to a round, distinct, well-defined hypoechoic solid mass with a thin hypoechoic circumference that appears to protrude from the cervix. With Doppler ultrasound imaging, the cervical leiomyoma appears with relatively poor perfusion compared to the surrounding normal myometrium. The imaging of a more hyperechogenic mass is usually associated with bleeding and degeneration of the cervical leiomyoma [14]. Also important is the ultrasound differential diagnosis of cervical leiomyomas from perivascular epithelioid cell tumors of the uterine cervix. In most cases of cervical perivascular epithelioid cell neoplasms, a single lesion originating from the cervix is found with normal margins, heterogeneous echogenicity, absence of acoustic shadowing, and presence of moderate to increased vasculature [15].

Magnetic resonance imaging (MRI), due to the higher resolution of soft tissues that characterizes it, is currently the best method for imaging the normal anatomy of the cervix and evaluating cervical lesions. Magnetic resonance imaging is estimated to be of significant help in the challenging differential diagnosis of cervical fibroids from adnexal tumors [16]. On MRI, cervical leiomyomas appear as a well-defined, rounded, T2 hypo-isointense lesion showing homogeneous enhancement centered on the cervix. Various types of cervical tumor degeneration can result in altered signal intensity [17]. However, in no case can ultrasound, computed tomography, or magnetic resonance imaging accurately differentiate leiomyoma from malignant tumors located in the uterine corpus or cervix [18]. In our patient, the preoperative imaging could not accurately establish the diagnosis of cervical leiomyoma. Its accurate differentiation from subserosal pedunculated uterine leiomyoma or solid adnexal mass with the help of ultrasonography and computed tomography was not possible. MRI was not available. The suspicion of cervical leiomyoma was a result of the imaging findings in combination with the partial deformation of the cervix, as found during the clinical examination. The diagnosis was established intraoperatively and confirmed by histological examination of the surgical specimen.

Surgery is the main treatment option for cervical leiomyomas. Performing hysterectomy or myomectomy with laparotomy or laparoscopy remains the cornerstone of the treatment approach for cervical leiomyomas and should be customized based on symptomatology, tumor size, patient age, and her desire to maintain fertility [19]. Surgical treatment of cervical fibroids is usually difficult. The risk of intraoperative bleeding and injury to the organs adjacent to the cervical leiomyoma is found to be increased, especially in cases of large fibroids, as in our case. The significant experience of surgeons and the appropriate planning of individual treatment centers should be included in the requirements for the surgical treatment of women with leiomyomas originating from the cervix [20]. Performing a total laparoscopic hysterectomy to treat cervical leiomyomas, although often leads to significant blood loss, since the perfusion of the cervix, including the uterine vessels, is increased when performed by surgeons with a high level of laparoscopic skill, the success rates are high and are accompanied by a low rate of complications [21,22]. Also, only a few cases of treatment of cervical leiomyomas with laparoscopically assisted vaginal hysterectomy have been reported in the literature [23,24].

The promising interventional radiology techniques in recent years for the treatment of uterine leiomyomas have not had the expected results [19]. Uterine artery embolization in patients with symptomatic cervical leiomyomas is an excellent alternative treatment option for those women who wish to preserve the uterus. Although the effectiveness of the method estimated to date remains poor, selective arterial embolization aims at the regression of the fibroids resulting in the reduction of pain, pressure symptoms, and bleeding to significantly improve the quality of life and achieve a future pregnancy. It is estimated that uterine artery embolization is a valuable therapeutic option for those women who are looking for a non-surgical choice for the treatment of cervical leiomyomas with a low rate of complications. Pain, exhaustion, nausea, vomiting, and vaginal discharge are all mild symptoms that may be observed after uterine artery embolization [25,26]. Similarly, hormonal therapy using a gonadotropin-releasing hormone agonist or a selective estrogen receptor modulator should be included in the conservative non-surgical treatment approach of patients with cervical leiomyomas [27].

## Conclusions

Large leiomyomas of the cervix are extremely uncommon, and their treatment with main laparotomy or laparoscopy surgery presents several difficulties and challenges. Chronic obstructive uropathy secondary to large cervical leiomyoma can result in renal dysfunction with a significantly increased risk of morbidity and mortality. Thorough clinical and imaging preoperative evaluation allows early diagnosis of hydroureteronephrosis and renal dysfunction-related chronic pelvic pain in women with cervical leiomyomas and aims to promptly implement the most appropriate modern available treatment options to avoid permanent renal damage and ensure the good health and quality of life of these patients.
